# On the Inhalation of Solid Medicines in Diseases of the Lungs and Trachea

**Published:** 1849-06

**Authors:** M. M. Rodgers

**Affiliations:** Rochester, N. Y.


ART. II.—*On the Inhalation of Solid Medicines in Diseases of the Lungs and Trachea.* By M. M. RODGERS, M. D., Rochester, N. Y.

The practice of inhaling vapors in pulmonary diseases is of ancient origin. Many different medicines, including some of the most powerful, as well as the most inert, have been inhaled in the forms of fumes and vapors. Among these, are the steam of water, vinegar, aromatic herbs; the gases, chlorine, nitrous acid, nitrous oxide; and recently, the vapors of ether and chloroform; also, the fumes of sulphur, tar, bitumen, assafœtida, opium, tobacco, iodine, mercury, turpentine and various resins, &c.

There can be no doubt of the beneficial effects of inhaled vapors in lung diseases. Some medicines, as iodine and mercury, produce their full and specific effects when inhaled, while others do not, simply because they are not taken unchanged and in substance into the lungs; but they are burnt, or otherwise changed, or destroyed, by the processes employed to volatilize or vaporize them.

The mucus membrane of the lungs, when diseased, would doubtless require to be treated on the same principles, and often in the same manner, as that of the mouth, nose, vagina, &c., if this surface were accessible to solid medicines, or fluid solutions. This inaccessibility is the reason why all our remedies in lung diseases must pass through the general circulation, before they reach the lungs. We are deprived of the advantage of local applications. This is one reason, also, why lung diseases are much more fatal than those of other organs. Now if these propositions are true, there is reason to hope for a great reform and improvement in our practice. We *can* make all parts of the lungs *accessible*, through the medium of the atmosphere; all medicinal substances which are capable of being reduced to fine, dry dust, can be conveyed into the lungs just as easily as vapors—or as fluid medicines can be taken into the stomach. And if we can diagnose correctly the various morbid conditions of the lungs, and then know the physiological, mechanical and chemical action of each medicine upon other like tissues, we can use them by inhalation, just as safely as we would apply them to an open ulcer, a tubercular gland, or an inflammation or catarrh of the throat, urethra or vagina.

The menstruum in which the medicines are to be administered, being atmospheric air, they will require to be in a state of the most minute pulverization,—an impalpable powder. No difficulty will exist in suspending those which are light and dry, sufficiently long to be inhaled; but such as are heavy, as calomel and antimony,—and those which are both heavy and moist, from their water of crystalization, cannot be so easily suspended unless mixed with some other dust which is lighter, and which will adhere to the particles of the former, and carry them up, so that both will float in the air till they are inhaled. The suspension of the medicinal dust could be easily accomplished by the aid of some apparatus which would keep the air in agitation in the place which contained the medicine, while the patient inhaled it from a mouth-piece, as in case of chloroform. The medicines which I would propose for trial in this way, are, euphorbium, bloodroot, charcoal, nitrate of silver, sulphate of zinc, acetate of zinc, acetate of lead, sulphate of iron, sulphate of copper, carbonate of iron, calomel, tartarized antimony, nitrate of potash, black sulphuret of mercury, iodide of potash, opium, ipecac, carbonate of lime, white sugar, lobelia, tobacco, kino, tannin, morphine, lupuline, licorice, and nitrate of bismuth. The diseases in which medication could thus be employed may be briefly indicated;—in chronic bronchitis and catarrhal inflammation of the air cells, nitrate of silver, acetate of lead and sulphate of zinc; in hæmoptysis, opium, tannin, kino, and acetate of lead; in acute inflammation, after expectoration has commenced, white sugar, licorice, morphine; in debilitated, scrofulous habits, carbonate of iron, acetate of zinc, iodide of potash; in dryness of the mucus surface, with tightness across the chest, nitrate of potash, lobelia and ipecac; in obstruction of the air cells and bronchiæ by viscid mucus, nitrate of potash, lobelia; in the formation of false membranes and cylinders of coagulable lymph, calomel and nitrate of potash; in spasmodic asthma and tickling cough, tobacco, morphine and lobelia; in the acute stage of inflammation, sulphate of iron and nitrate of potash. In proposing to the consideration of the profession, this mode of administering medicines, I would by no means discourage the use of steam and fumes of various substances; some of them might be employed alternately with the solid medicines, and others have sufficient value to entitle them to more general use and greater confidence, when used alone.

The utility of this practice is yet upon questionable ground, and must be established by actual experiment, if at all. There are several objections to this theory, which still appear, a priori, to be quite as hypothetical as the theory itself. The modus operandi of medicines can never be determined by the kind of reasoning adopted by the rationalists; in our reasoning on this subject, we must not draw conclusions from premises which appear merely *rational,* nor establish our practice upon the basis of *common sense,*—nor place implicit confidence in our *acute discernment and sound judgment.* In morals and metaphysics, this kind of logic will answer; but in demonstrative, physical science, it is calculated only to mislead the theorist, and lessen experimental research. The laws which govern a science of facts are too inflexible to bend to the dictates of rationalism. We must reason from a *known cause* to an *actual effect.* This theory, then, can be established as therapeutical science, only by *experiment.* I propose to pursue the study of this subject farther, and consider objections in another article.

